# Association of serum creatinine with aortic arch calcification in middle-aged and elderly adults: an observational cross-sectional study from China

**DOI:** 10.1186/s12872-022-02617-6

**Published:** 2022-04-12

**Authors:** Feifei Zhang, Nannan Hao, Lei Wang, Guoming Sun, Xiaoke Feng, Chunjian Li, Wenfeng Tan, Fang Wang

**Affiliations:** 1 Department of Cardiology, The First Affiliated Hospital of Nanjing Medical University, Nanjing, 210029 Jiangsu China; 2 Department of Rheumatology, The First Affiliated Hospital of Nanjing Medical University, Nanjing, 210029 Jiangsu China; 3 Division of Rheumatology, The Affiliated Changzhou NO.2 People’s Hospital of Nanjing Medical University, Changzhou, 213004 Jiangsu China; 4 Integrated Traditional Chinese and Western Medicine Institute of Nanjing Medical University, Nanjing, 210029 Jiangsu China

**Keywords:** Aortic arch calcification, Serum creatinine, Female

## Abstract

**Background and aims:**

Vascular calcification (VC) is a strong predictor of cardiovascular events and all-cause mortality in cardiovascular diseases (CVD). Renal dysfunction is closely related to VC. Serum creatinine, as an important indicator of renal function in chronic kidney disease (CKD), is closely associated with increased VC. Here, to explore the potential role of serum creatinine in CVD, we examined the association between serum creatinine level and aortic arch calcification (AAC) presence in a larger general population.

**Methods:**

A total of 9067 participants aged > 45 years were included in this study. All participants underwent postero-anterior chest X-ray examination to diagnose AAC. According to the distribution characteristics, serum creatinine levels in male and female were divided into tertiles respectively. Univariate and multivariate logistic regression analysis were used to analyze the association between aortic calcification and serum creatinine.

**Results:**

Participants included 3776 men and 5291 women, and 611 and 990 AAC were detected, respectively. Serum creatinine level in the female AAC group was significantly higher than that in the non-AAC group (*p* < 0.001), while there was no significant difference in male serum creatinine between the two groups (*p* = 0.241). After logistic regression analysis excluded confounding factors, with the first tertile of serum creatinine as the reference, multivariable-adjusted ORs and 95% CIs of the second and the highest tertile of female and male were 1.045 (0.856–1.276), 1.263 (1.036–1.539); 0.953 (0.761–1.193), 0.948 (0.741–1.198), respectively.

**Conclusion:**

Elevated serum creatinine levels are independently associated with higher AAC incidence in female aged > 45 years old. Measuring serum creatinine levels may assist the early screening individuals at high risk of developing CVD. And higher attention should be given to female's serum creatinine levels in daily clinical practice.

**Supplementary Information:**

The online version contains supplementary material available at 10.1186/s12872-022-02617-6.

## Introduction

Vascular calcification (VC) was initially thought to be a passive, degenerative process and regarded as part of the normal aging process [1]. However, evidence now suggests VC involves complex pathophysiological processes in patients with cardiovascular disease (CVD) resulting from diabetes, hypertension, atherosclerosis and chronic kidney disease (CKD) [2]. Moreover, VC has been reported involvedin nearly every part of aorta, from the aortic arch, to the aortic valves and also in the abdominal aorta. Although the specific mechanism responsible for VC in aorta and the impairment of cardiovascular functions has not been fully understood, accumulating evidence suggests VC is an independent risk factor for cardiovascular events and all-cause mortality in CVD [3–5]. Because no therapies are currently available to prevent or reverse aorta calcification, recently new therapeutic strategies to diagnose or prevent the onset of the VC process are highlighted [6]. Therefore, identification of novel, promising and clinically feasible markers that could assist early warning risk is urgently needed, especially in general population.

Massing evidence proved patients with kidney disease are prone to VC [7, 8], disorders of mineral metabolism (abnormal levels of serum calcium and phosphorus) and/or other potential uremic toxins may play a prominent role in the pathogenesis of VC in CKD. Creatinine is the end product of creatine phosphate metabolism, and creatine is a nitrogenous organic acid that is generated predominantly in the kidney and liver [9, 10]. It is widely accepted that serum creatinine is excreted mainly by the kidney and is the most commonly used biomarker of kidney function [11]. In chronic kidney disease populations, the simultaneous assessment of creatinine—based eGFR and albumin—to—creatinine ratio will facilitate the improved cardiovascular risk classification [12]. However, the association of serum creatinine level and VC presence is sparsely investigated and needs more evidence. In addition to kidney disease, there have been several studies that showed serum creatinine is closely associated with aorta calcification in multiple chronic diseases. In long-standing type 2 diabetes, the albumin-to-creatinine ratio predicted progression of coronary artery calcification [13]. Moreover, some studies have shown aortic calcification score is strongly associated with serum creatinine concentration in type 2 diabetic patients [14]. Thus, it is reasonable to speculate that serum creatinine level might be closely related to VC process.

The variation of serum creatinine level trends to diseases with age or gender. In addition to kidney disease or above-mentioned chronic diseases, whether creatinine levels affect vascular calcification in the general population is unknown. To better explore the relationship of serum creatinine and VC, we therefore conducted a cross-sectional analysis in a larger general population to investigate the association between serum creatinine and aorta calcification presence. Due to aortic arch calcification (AAC) is one of the easily identifiable type of aorta calcification, and is highly prevalent detected by chest X-ray in the general population [15], we employed AAC to evaluate the VC presence in this study. Our present data provide evidences that serum creatinine is highly associated with VC occurrence rate in female aged > 45 years old.

## Methods

### Study population

**Fig. 1:**
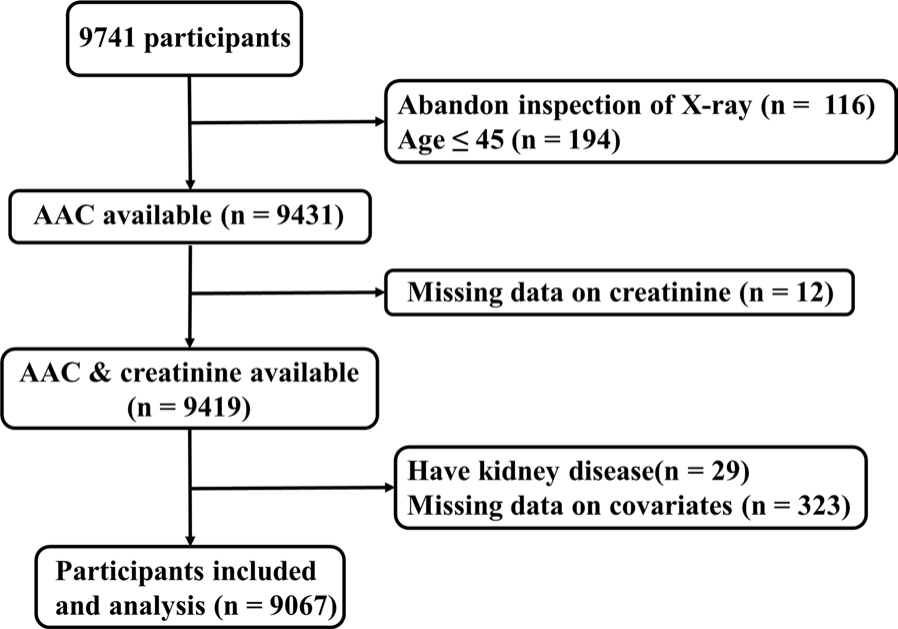
Flow of participants

Totally 9741 participants were recruited from health examination program of Qinglong Community of Changzhou city, Jiangsu Province, China from January 2019 to December 2020. Participants were eligible for inclusion if they (1) aged > 45 years old, (2) complete clinical data, (3) without prior self-reported kidney disease. Finally, a total of 9067 participants were included in this cross-sectional study. Flow chart of participants in recruitment for the study in shown in Fig. 1.

### Aortic arch calcification (AAC) assessment

AAC was assessed using postero-anterior chest X-ray examination (Multix Select DR, Siemens, Germany). AAC evaluation was executed on the basis of the image in chest X-ray examination on an electronic medical record by trained radiologists blinded to each participant’s information.

### Data collection

Data regarding the presence of hypertension, coronary heart disease (CHD), and diabetes mellitus were recorded by an experienced physician. CHD was defined as self-reported physician-diagnosed CHD. Hypertension was defined as self-reported physician-diagnosed hypertension, taking antihypertensive medications, or having a systolic blood pressure of ≥ 140 mm Hg and/or diastolic blood pressure of ≥ 90 mm Hg [16]. Diabetes mellitus was defined as self-reported physician-diagnosed diabetes, taking oral hypoglycemic agents or insulin, a fasting glucose level ≥ 126 mg/dL, or a plasma glucose level ≥ 200 mg/dL 2 h after oral glucose tolerance test [17]. Anthropometric parameters were measured in the standing position with patients wearing light indoor clothing and no shoes. BMI was calculated as weight (kg) divided by height (m)^2^. Blood pressure was measured using an automatic manometer after the patient rested for at least 5 min.

### Laboratory measurements

Blood samples were obtained from an antecubital vein after a minimum 8 h fasting period. Serum was collected after centrifuge at 3000 rpm for 10 min, and serum glucose, creatinine, urea nitrogen, urea acid, triglyceride, total cholesterol, HDL cholesterol, LDL cholesterol were measured using a fully automatic chemistry analyzers (FACA-200, Thomas Scientific, USA).

### Statistical analysis

Statistical analyses were performed using SPSS version 23.0 and GraphPad Prism 8.0.2 software. First, all of the participants were divided into aortic arch calcification group and non- arch calcification group. Then we divided the creatinine level into three tertiles to study the association between AAC and creatinine separately in male and female. Continuous variables were expressed as mean ± standard (mean ± SD), and categorical data were presented as percentages. Differences in continuous variables between groups were assessed by unpaired two-tailed *t*-test and analysis of variance (ANOVA). Categorical data and proportions were analyzed by chi-square test. Logistic regression analysis (univariate and multivariate) was performed to determine the influencing factors of aortic arch calcification. Potential confounding factors included age, sex, hypertension, Diabetes mellitus, CHD, serum glucose, uric acid, serum total cholesterol and HDL cholesterol. Odds ratio (ORs) and corresponding 95% confidence intervals were reported. *P* < 0.05 was considered statistically significant.

## Results

### Characteristics of participants

A total of 9067 participants were included in this study. There were 3776 (41.6%) male and mean age was 66.31 ± 8.63 years. The prevalence of AAC was 17.6% in the entire participants, with more than half had hypertension (59.2%), 15.6% had diabetes mellitus and 1.6% had CHD. We divided the participants into two groups of male and female to further analyze the link with AAC (Table 1). Regardless of males and females, the incidence of hypertension, diabetes mellitus and CHD in the AAC group was higher than that in the non-AAC group. Serum glucose, serum creatinine and uric acid among female had significant increase between non-AAC and AAC, while there was no significant difference in male.

**Table 1:** Characteristics of the study participants

Index	All (n = 9067)	Female (n = 5291)			Male (n = 3776)		
		Without AAC (n = 4301)	With AAC (n = 990)	*P* value	Without AAC (n = 3165)	With AAC (n = 611)	*P* value
Age (years)	66.31 ± 8.63	63.60 ± 8.45	72.99 ± 6.91	< 0.001	66.85 ± 7.85	71.82 ± 7.16	< 0.001
BMI (kg/m^2^)	24.78 ± 3.42	24.82 ± 3.58	24.85 ± 3.43	0.793	24.73 ± 3.21	24.70 ± 3.32	0.815
SBP (mmHg)	138.17 ± 19.47	136.86 ± 19.51	144.29 ± 19.86	< 0.001	137.36 ± 18.90	141.69 ± 19.23	< 0.001
DBP (mmHg)	79.58 ± 10.86	78.49 ± 10.69	76.56 ± 10.24	< 0.001	81.88 ± 10.75	80.23 ± 11.24	0.001
Diabetes mellitus	1415 (15.6%)	605 (14.1%)	214 (21.6%)	< 0.001	472 (14.9%)	124 (20.3%)	0.001
Hypertension	5371 (59.2%)	2229 (51.8%)	690 (69.7%)	< 0.001	2029 (64.1%)	423 (69.2%)	0.015
Coronary heart disease	146 (1.6%)	37 (0.9%)	25 (2.5%)	< 0.001	63 (2.0%)	21 (3.4%)	0.026
Serum glucose (mmol/L)	6.48 ± 1.81	6.39 ± 1.77	6.67 ± 1.93	< 0.001	6.49 ± 1.77	6.68 ± 2.04	0.015
Serum creatinine (µmol/L)	71.32 ± 22.21	62.09 ± 17.88	68.37 ± 22.91	< 0.001	82.43 ± 21.67	83.53 ± 19.70	0.241
Serum urea nitrogen (mmol/L)	5.13 ± 1.58	5.09 ± 1.50	5.02 ± 1.68	0.227	5.22 ± 1.65	5.11 ± 1.54	0.101
Serum uric acid (µmol/L)	291.91 ± 94.58	256.54 ± 79.28	278.60 ± 87.33	< 0.001	334.46 ± 93.89	342.10 ± 97.59	0.068
Serum total cholesterol (mmol/L)	5.05 ± 1.15	5.31 ± 1.13	5.14 ± 1.12	< 0.001	4.74 ± 1.07	4.72 ± 1.19	0.572
Serum triglycerides (mmol/L)	1.73 ± 1.25	1.78 ± 1.23	1.80 ± 1.36	0.72	1.66 ± 1.24	1.60 ± 1.17	0.284
Serum HDL cholesterol (mmol/L)	1.34 ± 0.37	1.39 ± 0.36	1.36 ± 0.36	0.009	1.26 ± 0.36	1.28 ± 0.36	0.301
Serum LDL cholesterol (mmol/L)	2.85 ± 0.77	3.02 ± 0.76	2.97 ± 0.78	0.072	2.64 ± 0.73	2.63 ± 0.77	0.670

AAC: Aortic arch calcification

### Comorbidity distribution in creatinine tertiles in female

**Fig. 2:**
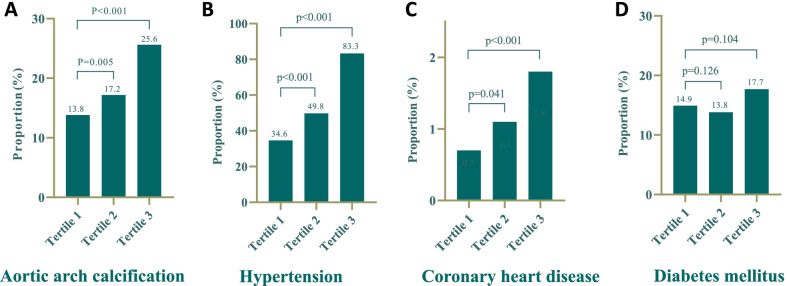
Prevalence of AAC (**A**), hypertension (**B**), coronary heart disease (**C**) and diabetes mellitus (**D**) according to the serum creatinine tertiles among female

In order to investigate the role of creatinine for AAC in female, serum creatinine levels were divided into tertiles. We found that with the level of creatinine elevated, the incidence of AAC was significantly increased (Tertile1 to Tertile3 were 13.8%, 17.2%, 25.6%, respectively; *p* < 0.001 for trend) (Fig. 2A). In addition, the prevalence of hypertension (Tertile1 to Tertile3 were 34.6%, 49.8%, 83.3%, respectively; *p* < 0.001 for trend) (Fig. 2B) and CHD (Tertile1 to Tertile3 were 0.7%, 1.1%, 1.8%, respectively; *p* < 0.001 for trend) (Fig. 2C) also increased accompanied by creatinine tertiles, while the prevalence of diabetes mellitus had no significance difference with creatinine tertiles (*p* = 0.104 for trend) (Fig. 2D).

### Association of serum creatinine with AAC in female

Based on the variables with *p* < 0.05 shown in Table 1, we performed univariate logistic regression analysis and found that age, diabetes mellitus, hypertension, CHD, serum glucose and uric acid were risk factors for AAC in female. Uric acid and high cholesterol were not significantly associated with AAC in male (Table 2).

**Table 2:** Univariate logistic regression analysis of AAC in general population

Parameters	Female		Male	
	OR (95% CI)	*P* value	OR	*P* value
Age (years)	1.147 (1.135–1.158)	< 0.001	1.086 (1.074–1.099)	< 0.001
*Sex*				
SBP (mmHg)	1.019 (1.015–1.022)	< 0.001	1.012 (1.007–1.016)	< 0.001
DBP (mmHg)	0.983 (0.976–0.989)	< 0.001	0.986 (0.978–0.994)	0.001
Diabetes mellitus	1.684 (1.415–2.004)	< 0.001	1.454 (1.167–1.812)	0.001
Hypertension	2.138 (1.844–2.480)	< 0.001	1.260 (1.045–1.518)	0.015
Coronary heart disease	2.985 (1.788–4.981)	< 0.001	1.754 (1.062–2.897)	0.028
Serum glucose (mmol/L)	1.079 (1.043–1.117)	< 0.001	1.055 (1.010–1.103)	0.016
Serum urea nitrogen (mmol/L)	0.972 (0.928–1.018)	0.227	0.955 (0.904–1.009)	0.101
Serum uric acid (µmol/L)	1.003 (1.002–1.004)	< 0.001	1.001 (1.000–1.002)	0.068
Serum total cholesterol (mmol/L)	0.874 (0.820–0.931)	< 0.001	0.977 (0.902–1.058)	0.580
Serum HDL cholesterol (mmol/L)	0.772 (0.635–0.937)	0.009	1.133 (0.894–1.435)	0.301

AAC: Aortic arch calcification

**Fig. 3:**
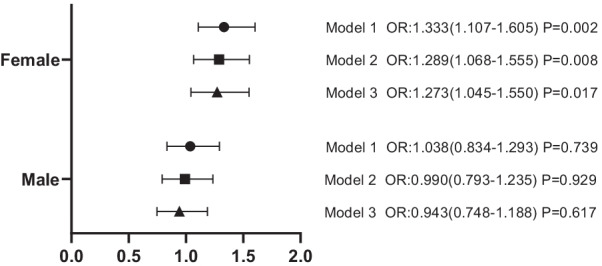
Association of serum creatinine and AAC. ORs for AAC associated with the highest tertile compared with the lowest tertile. Model 1 adjusted for and age. Model 2 further adjusted for diabetes mellitus, hypertension and coronary heart disease. Model 3 further adjusted for serum uric acid and serum glucose

We subsequently conducted multiple logistic regression analyses to adjusted conventional cardiovascular risk factors. Compared with participants in the lowest tertile, the unadjusted odds ratio (OR) for the presence of AAC in the highest creatinine tertile was 1.193 (95% confidence interval [CI], 0.966–1.474; *p* = 0.095 for trend) in male and 2.144 (95% CI: 1.811–2.539; *p* < 0.001 for trend) in female. After adjusting for several covariates including age, hypertension, diabetes mellitus, CHD, serum glucose and uric acid, we found the association between serum creatinine and AAC among female remained statistically significant in all multivariate logistic regression models (Additional file 1: Table S1). Compared with participants in the first tertile of creatinine, those in the third tertile had higher odds for AAC in all models (unadjusted model: OR = 2.144, 95% CI: 1.811–2.539; model 1: OR = 1.333, 95% CI: 1.107–1.605; model 2: OR = 1.289, 95% CI: 1.068–1.555; model 3: OR = 1.272, 95% CI: (1.044–1.549) (Fig. 3). In all models, there is no significant association between AAC and serum creatinine in male.

## Discussion

In present study, we investigated the association of serum creatinine levels with prevalence of aortic arch calcification (AAC) in a larger middle-aged and elderly Chinese population. Our data showed that higher serum creatinine levels were positively correlated with the increased AAC prevalence, importantly, the significant correlation was only showed in female.

These results have important clinical significance. First, AAC is a subclinical feature of CVD. Our data provide a novel approach to evaluate AAC risk in general population. We proposed that individuals with high serum creatinine level may need routine monitoring of heart health that may prevent their future CVD risk. Second, serum creatinine level in addition to traditional CVD risk factors should be taken into account when evaluating risk for development of CVD in in middle-aged and elderly adults, especially in female.

Multiple studies have demonstrated that age and gender are two independent risk factors for CVD. The severity of CVD is associated with older age and burden of CVD risk factors including hypertension, diabetes, and obesity [18, 19]. More importantly, it is reported that female with CVD risk factors, such as hypertension, are more likely to develop vascular stiffness and VC process than male under similar risks [20], consisting with our findings, but without further evidence for the cause. In this cohort study, the age of all participants are more than 45 years old, therefore most of female were in middle to older age or in menopause. Another Chinese study came to similar conclusions to ours that metabolic syndrome in postmenopausal women is associated with a higher prevalence of CAD than in premenopausal women and men with metabolic abnormalities [21]. Our results show that the prevalence of AAC in female (18.7%) is higher than that in male (16.2%), and age is an independent risk factor for AAC. These findings suggest that in older age, compared with males, females are more likely to have VC, a manifestation of preclinical coronary atherosclerosis or CVD.

Serum creatinine is generally used as a functional biomarker of renal function. Recently, more and more evidence show that serum creatinine level is positively associated with the prevalence of aorta or coronary artery calcification in CKD, diabetes patients or other renal disorders [22]. Considering prevalence of AAC is higher in female than that in male is observed in this study, we suspected that serum creatinine level might help to reflect the development to the AAC or CVD, especially in combination of other traditional risk factors including gender, age, and hypertension. Serum creatinine levels can be affected by non-renal factors, such as muscle mass, age, and sex. In our cohort, after adjusting for age, serum creatinine concentration of all males (83.60 ± 20.36 µmol/L) was higher than in all females (63.27 ± 19.08 µmol/L) aged > 45 years old, which is consistent with other published reports in older population [23]. It is unexpected that, serum creatinine level in gender difference has been demonstrated in healthy children and adults, which is explained by the greater increase in muscle mass in male than that in female [24]. Importantly, we observed after adjusting for the risk factor of age, serum creatinine level of female in the highest tertile was independently associated with AAC. Calcification of the vessel intima and media is associated with arterial stiffening and is a major cause of hypertension and CHD in the elderly [25]. In fact, our study also showed that the prevalence of hypertension and CHD also significantly increased accompanied by serum creatinine tertiles in female, in spite of the present data can’t exclude the confounding factors with mediation such as antihyperlipidemic or antihypertensive drugs. Taken together, these results suggest that female in older age with high serum creatinine level were at a higher risk for CVD, and serum creatinine have more prognostic values in female than in male for CVD risk evaluation.

Although we could not elucidate why serum creatinine was higher in older female with AAC than in those without AAC, it might be explained by the fact that sex hormone changes in older female, especially in postmenopausal women, could alter serum creatinine metabolism by reducing its degradation. A recent study has showed that increased body fat in postmenopausal female may alter serum creatinine levels [26]. Given the potential role of estrogen in the regulation of VC process [27], it is not surprised, that elderly female with high tertile of creatinine are more likely to have AAC. On the other hand, creatinine and its degradation products, such as creatol and methylguanidine, were also found in serum, uremia and/or inflamed tissues in CKD rats and thus likely to be pathological and further disease progression [9]. Therefore, the accumulative degradation products of creatinine in serum and/or vessel in older female with lower creatinine degradation activity may contribute to the VC process indirectly, suggesting female in older age with high serum creatinine are prone to be with AAC occurrence. Further studies are needed to elucidate the causative mechanism between serum creatinine and AAC.

In spite of the interesting finding, this study has some limitations. First, we can not obtain more serological indicators due to the limited physical examination items from the medical examination center. Second, chest X-ray examination is more practicable in large population screening than computer tomography, whereas, it can only perform qualitative rather than quantitative analysis for AAC. Third, since our research is a retrospective study, it lacks the detailed information on medication and the clear exclusion criteria of the participants, as a result, some confounding factors are included in the analysis. Further study is necessary to explore the causal relationship between serum creatinine and VC, demonstrating the feasibility of serum creatinine as a biomarker for VC and its pathological role in VC process.

## Conclusion

In conclusion, our data suggested serum creatinine levels are significantly associated with AAC in female aged > 45 years old. These results indicated that the role of serum creatinine levels in AAC and CVD development remains underrecognised and undertreated in the general population. Thus, AAC or asymptomatic CVD risk screening should be given for female with increased serum creatinine levels, especially in individuals concomitant with other CVD risk factors.

## Supplementary Information

**Additional file 1**. Association of AAC with creatinine tertiles.

## Data Availability

The datasets analyzed during the current study are not publicly available due to inclusion of other unpublished content but are available from the corresponding author on reasonable request.
