# Supplementary material for: Association of serum creatinine with aortic arch calcification in middle-aged and elderly adults: an observational cross-sectional study from China
Source: BMC Cardiovasc Disord. 2022 Apr 12;22:167. doi: 10.1186/s12872-022-02617-6 (PMC9004043; doi:10.1186/s12872-022-02617-6)
Supplement: Supplementary file 1 — Additional file 1. Association of AAC with creatinine tertiles. [file 12872_2022_2617_MOESM1_ESM.docx]

**Table S1** Association of serum creatinine with AAC in female.

| Serum Creatinine | Unadjusted | Model 1 | Model 2 | Model 3 |
| --- | --- | --- | --- | --- |
|  | OR (95% CI) | OR (95% CI) | OR (95% CI) | OR (95% CI) |
| Male |  |  |  |  |
| Tertile 1 | 1 (reference) | 1 (reference) | 1 (reference) | 1 (reference) |
| Tertile 2 | 1.050 (0.847-1.302) | 0.991 (0.795-1.236) | 0.972 (0.778-1.214) | 0.949 (0.758-1.187) |
| Tertile 3 | 1.193 (0.966-1.474) | 1.038 (0.834-1.293) | 0.990 (0.793-1.235) | 0.943 (0.748-1.188) |
| p for trend | 0.095 | 0.716 | 0.95 | 0.620 |
| Female |  |  |  |  |
| Tertile 1 | 1 (reference) | 1 (reference) | 1 (reference) | 1 (reference) |
| Tertile 2 | 1.297 (1.081-1.556) | 1.057 (0.868-1.287) | 1.048 (0.860-1.278) | 1.046 (0.857-1.276) |
| Tertile 3 | 2.144 (1.811-2.539) | 1.333 (1.107-1.605) | 1.289 (1.068-1.555) | 1.273 (1.045-1.550) |
| p for trend | <0.001 | 0.002 | 0.007 | 0.014 |

Model 1 adjusted for and age.

Model 2 further adjusted for diabetes mellitus, hypertension and coronary heart disease.

Model 3 further adjusted for serum uric acid and serum glucose
